# Performance of lipid fingerprint-based MALDI-ToF for the diagnosis of mycobacterial infections

**DOI:** 10.1016/j.cmi.2020.08.027

**Published:** 2021-06

**Authors:** Ximena Gonzalo, Agnieszka Broda, Francis Drobniewski, Gerald Larrouy-Maumus

**Affiliations:** 1) Department of Infectious Diseases, Faculty of Medicine, Imperial College London, London, W12 ONN, UK; 2) MRC Centre for Molecular Bacteriology and Infection, Department of Life Sciences, Faculty of Natural Sciences, Imperial College London, London, SW7 2AZ, UK

**Keywords:** Diagnostics, Evaluation, Lipids, MALDI, Mycobacteria

## Abstract

**Objectives:**

Bacterial diagnosis of mycobacteria is often challenging because of the variability of the sensitivity and specificity of the assay used, and it can be expensive to perform accurately. Although matrix-assisted laser desorption/ionization mass spectrometry (MALDI MS) has become the workhorse of clinical laboratories, the current MALDI methodology (which is based on cytosolic protein profiling) for mycobacteria is still challenging due to the number of steps involved (up to seven) and potential biosafety concerns. Knowing that mycobacteria produce surface-exposed species-specific lipids, we here hypothesized that the detection of those molecules could offer a rapid, reproducible and robust method for mycobacterial identification.

**Methods:**

We evaluated the performance of an alternative methodology based on characterized species-specific lipid profiling of intact bacteria, without any sample preparation, by MALDI MS; it uses MALDI-time-of-flight (ToF) MS combined with a specific matrix (super-2,5-dihydroxybenzoic acid solubilized in an apolar solvent system) to analyse lipids of intact heat-inactivated mycobacteria. Cultured mycobacteria are heat-inactivated and loaded directly onto the MALDI target followed by addition of the matrix. Acquisition of the data is done in both positive and negative ion modes. Blinded studies were performed using 273 mycobacterial strains comprising both the *Mycobacterium tuberculosis* (Mtb) complex and non-tuberculous mycobacteria (NTMs) subcultured in Middlebrook 7H9 media supplemented with 10% OADC (oleic acid/dextrose/catalase) growth supplement and incubated for up to 2 weeks at 37°C.

**Results:**

The method we have developed is fast (<10 mins) and highly sensitive (<1000 bacteria required); 96.7% of the Mtb complex strains (204/211) were correctly assigned as MTB complex and 91.7% (22/24) NTM species were correctly assigned based only on intact bacteria species-specific lipid profiling by MALDI-ToF MS.

**Conclusions:**

Intact bacterial lipid profiling provides a biosafe and unique route for rapid and accurate mycobacterial identification.

## Graphical abstract

**Figure:**
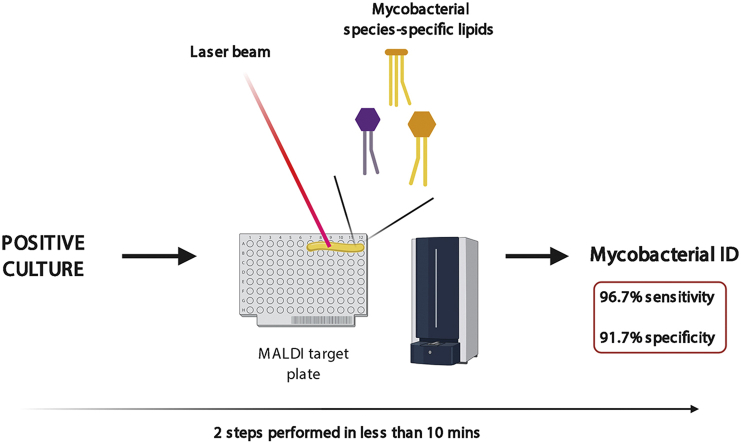

## Introduction

Tuberculosis is one of the biggest infectious disease killers in the world, remaining in the top ten causes of mortality worldwide [1]. Control of the disease is hampered by multiple factors, including the difficulties in getting a timely and accurate diagnosis, a key pillar in the WHO End TB Strategy [2]. In the last few decades, matrix-assisted laser desorption/ionization time-of-flight (MALDI-ToF) has been implemented in the general bacteriology laboratory, revolutionizing bacterial identification; it has also been useful in the identification of mycobacteria and antibiotic resistance [3,4]. However, its broad application for mycobacteria has been challenging due to the fact that sample preparation and handling should happen in biosafety level 3 facilities, and that the mycobacterial wall needs special treatment to allow the molecules of interest to be released for MALDI-ToF proteomic analysis. This is time-consuming and requires a careful extraction step [5]. A novel approach has been described recently which aims to identify *Mycobacterium tuberculosis* based on MALDI-ToF mass spectrometry (MS) analysis of lipids from intact bacteria that would overcome, at least partially, the issues stated above [6].

The mycobacterial wall is composed of 60% lipids such as C-mycoside glycopeptolipid, phenol glycosides, trehalose-containing lipopolysaccharides, sulpholipids, lipoarabinomannan, and mycolic acids [7]. The plasma membrane, the innermost layer, consists of a lipid bilayer interacting with proteins, peptidoglycan, arabinogalactan and polysaccharides esterified with mycolic acids. The free lipids associated with the outer membrane are very complex and structurally diverse [8]. Some lipids—such as sulphoglycolipids (SL-I) and the polyacyltrehaloses (PATs)—are specific for the *M. tuberculosis* complex; other lipids, such as C-mycoside glycopeptidolipids (GPLs), are found only in non-tuberculous mycobacteria (NTM) (e.g. *M*. *avium*) [9,10].

A simple procedure which can identify Mtb and drug resistance quickly and cheaply using standard commercially available MALDI-ToF instruments in clinical microbiology laboratories would be a significant asset in relation to turn-around times, enabling more timely clinical decision making.

The aim of this study was to establish a methodology to differentiate *M. tuberculosis* isolates from the NTM group and within NTM species based on species-specific lipid profiling using lipid fingerprints in MALDI-ToF MS on intact bacteria.

## Material and methods

### Clinical *Mycobacterium tuberculosis* isolates

A total of 273 mycobacterial isolates archived at Imperial College, London, UK were cultured from frozen aliquots into Middlebrook 7H9 broth supplemented with OACD (oleic acid/dextrose/catalase) without glycerol (Sigma-Aldrich Company Ltd, Gillingham, Dorset, UK) and incubated for 4–6 weeks at 37°C. The isolates comprised 244 Mtb isolates of different lineages (Beijing, Ural, Ghana-like1, Ghana-like2, X-like, EAJ, Haarlem, LAM) and 29 NTM isolates including 17 species (*M*. *abscessus*, *M*. *avium* (x2), *M*. *bohemicum* (x2), *M*. *celatum* (x2), *M*. *fortuitum* (x2), *M. interjectum*, *M*. *intracellulare*, *M*. *kansasii*, *M*. *marinum* (x2), *M*. *mucogenicum* (x2), *M*. *nonchromogenicum* (x2), *M*. *peregrinum* (x2), *M*. *phlei* (x2), *M*. *shimoidei* (x2), *M*. *simiae* (x2), *M*. *smegmatis* (x2), and *M*. *xenopi*. Mtb cultures were originally isolated from sputa of patients with pulmonary *M. tuberculosis* infections and whole-genome sequencing (WGS) was obtained for all of them. A collection of NTM reference strains was obtained from the European Centre for Disease Prevention and Control (ECDC) Reference Laboratory Network.

### Sample preparation

Once optimal growth of the bacteria was obtained, 100 μL of bacterial suspension were placed into 1.5 mL Eppendorf tubes and heat-killed at 95°C for 30 min before leaving the BSL3 containment area. The heat-killed Mtb and NTM pellets were washed four times with 200 μL of double-distilled water. Then, a 0.4 μL aliquot of the resuspended pellet was pipetted onto the MALDI matrix plate and mixed with 0.8 μL of the MALDI matrix. The matrix used consisted of a 9:1 mixture of 2,5-dihydroxybenzoic acid and 2-hydroxy-5-methoxybenzoic acid (super-DHB, Sigma-Aldrich) at a concentration of 10 mg/mL in chloroform:methanol 9:1. (Supplementary Material Fig. S1). All solvent manipulations and handling were carried out under a fume hood.

### Mass spectrometry analysis

An Applied Biosystems 4800 MALDI TOF/TOF™Analyzer (Applied Biosystems/MDS SCIEX MDS Sciex, Concord, Ontario, Canada) was used. Samples were analysed in the positive and negative ion mode operating at 20 kV and were set to acquire mass spectral peaks with mass/charge ratio (*m*/*z*) from 400 to 4000 mass units. Mass spectrometry data were analysed using Data Explorer® Software version 4.9 from Applied Biosystems and assignment of Mtb/NTM was performed blinded, after visual interpretation of spectra following criteria previously published [10]. Two people performed the visual assessment independently.

### Statistical analysis

Sensitivity, specificity and 95% confidence intervals were calculated as described in the literature [[11], [12], [13], [14]].

## Results

The study examined the lipidomic profile of the TB isolates by MALDI-ToF mass spectrometry. In total, 273 isolates were tested (244 Mtb isolates and 29 NTM isolates). The mass spectra were acquired in both positive and negative mode.

Analysis of the mass spectra was conducted blindly. A good mycobacterial mass spectral signal (resolution >300 and signal-to-noise >5) in negative mode was generated for 235 out of 273 isolates; 38 isolates (14%) could not be assigned to either the Mtb or the NTM group, and nine isolates (3%) were misidentified. Good signals (resolution >300 and signal-to-noise >5) for most of the NTMs were generated in positive and negative ion modes. The 226 isolates of 235 which were correctly assigned (Table 1) were based on the negative mode signature for Mtb and the positive mode signatures for NTMs. All data were compared to molecular identification and reference strains inventory designation from the ECDC European Reference Laboratory Network. Identification of Mtb was achieved by the presence of SL-I, which is a negatively charged lipid uniquely found in Mtb [15,16]. Within the NTM group (29 clinical isolates), identification was performed in the positive ion mode by the ionization of species-specific lipid fingerprint—such as glycopeptidolipids, C-mycosides, polar glycopeptidolipids, glycerol monomycolate and phenolic glycolipids [[17], [18], [19], [20], [21], [22], [23], [24], [25], [26], [27], [28]]—associated to each of the species exhibiting distinct and unique signatures or ‘chemical barcoding’, allowing clear discrimination of the NTM clinical isolates from each other presented in this study (Supplementary Material Fig. S2).

**Table 1:** Sensitivity and specificity of the lipid fingerprint-based matrix-assisted laser desorption/ionization time-of-flight (MALDI-ToF) method

Mycobacterial identification (*n* = 235)		WGS		Sensitivity (%)	Specificity (%)
		Positive	Negative		
MALDI-ToF	Positive	204	2	96.7	91.7
	Negative	**7**	**22**		

WGS, whole-genome sequencing.

Nine isolates (3%) gave discrepant results compared to molecular DNA-derived identification and/or other biochemical analysis underpinning the designation by the ECDC European Reference Laboratory network; 7/211 Mtb strains were wrongly identified as NTMs and 2/24 NTMs (*M. nonchromogenicum* and *M. smegmatis*) were assigned as Mtbs by MALDI-ToF. The 38/273 uninterpretable isolates (14%) were tested twice from the same heat-killed washed pellets. The results remained the same, i.e. uninterpretable. Of those 38 isolates, 33 were Mtb and five NTM isolates. Regarding the five NTM isolates—*M. abscessus*, *M. mucogenicum*, *M. phlei*, *M. shimoidei* (x2)—the quality of the spectra was poor associated with a low signal-to-noise (<5) precluding a convincing assignment of the data generated. For the 33 Mtb isolates (distributed across Beijing, Ghana-like 1, Latín American Mediterranean and clade A groups), as our method relies mainly on the presence of SL-I, failure to assign these as Mtb strains could be explained by the low abundance of SL-I in those strains, i.e. below the limit of detection. It has been reported in the literature that some Mtb strains within the same Mtb lineages can have low or no detectable SL-I [29]. Therefore, variations in the content of sulphatides can explain the un-interpretability of the 33 Mtb isolates, and further work needs to be carried out in order to confirm or refute this hypothesis.

The sensitivity and specificity of the MALDI-ToF (excluding uninterpretable results) was 96.7% (204/211; 95%CI 93.3–98.5%) and 91.7% (22/24; 95%CI 73.0–98.9%) respectively (Table 1).

## Discussion

Current proteomic approaches require several extraction steps and incubation times for disruption of the bacteria in order to gain access to intracellular proteins [[30], [31], [32], [33], [34]]. By contrast, the lipidomics approach allows the identification of surface-exposed species-specific lipids, and is performed in a two-step process, making workflow in the laboratory very straightforward (Supplementary Material Table S1).

MALDI-ToF MS using an intact bacterial lipidomics approach generated high-quality interpretable spectra for 86% of the samples tested (235/273), with a sensitivity (96.7%; 204/211)—very similar to that of the quickest proteomics approach on MALDI-ToF that identified 91.7% at species/complex level and an additional 4.4% to genus level [35]—but with the advantage of a single processing step after heat inactivation (Supplementary Material Table S1). Furthermore the samples were heat-treated pre-analysis, rendering the Mtb cultures safe for analysis.

Phosphatidylinositol mannosides (PIMs), sulpholipids (SL-I) and NTM species-specific lipids were easily recognized visually using this method, allowing for genus [36] and Mtb complex identification respectively. When the spectra were poor quality, non-genus-level identification was still possible. Failure to produce a good quality signal might be due to poor sample quality. It was not related specifically to NTM or Mtb cultures. The sensitivity and specificity for isolates for which a spectrum was identifiable was 96.7% (204/211) and 91.7% (22/24), respectively. Such percentages are higher than those found when using a protein-based approach [32,[37], [38], [39], [40]], suggesting that the lipid-based approach is a very promising tool for the identification of mycobacteria by MALDI-ToF.

**Fig. 1:**
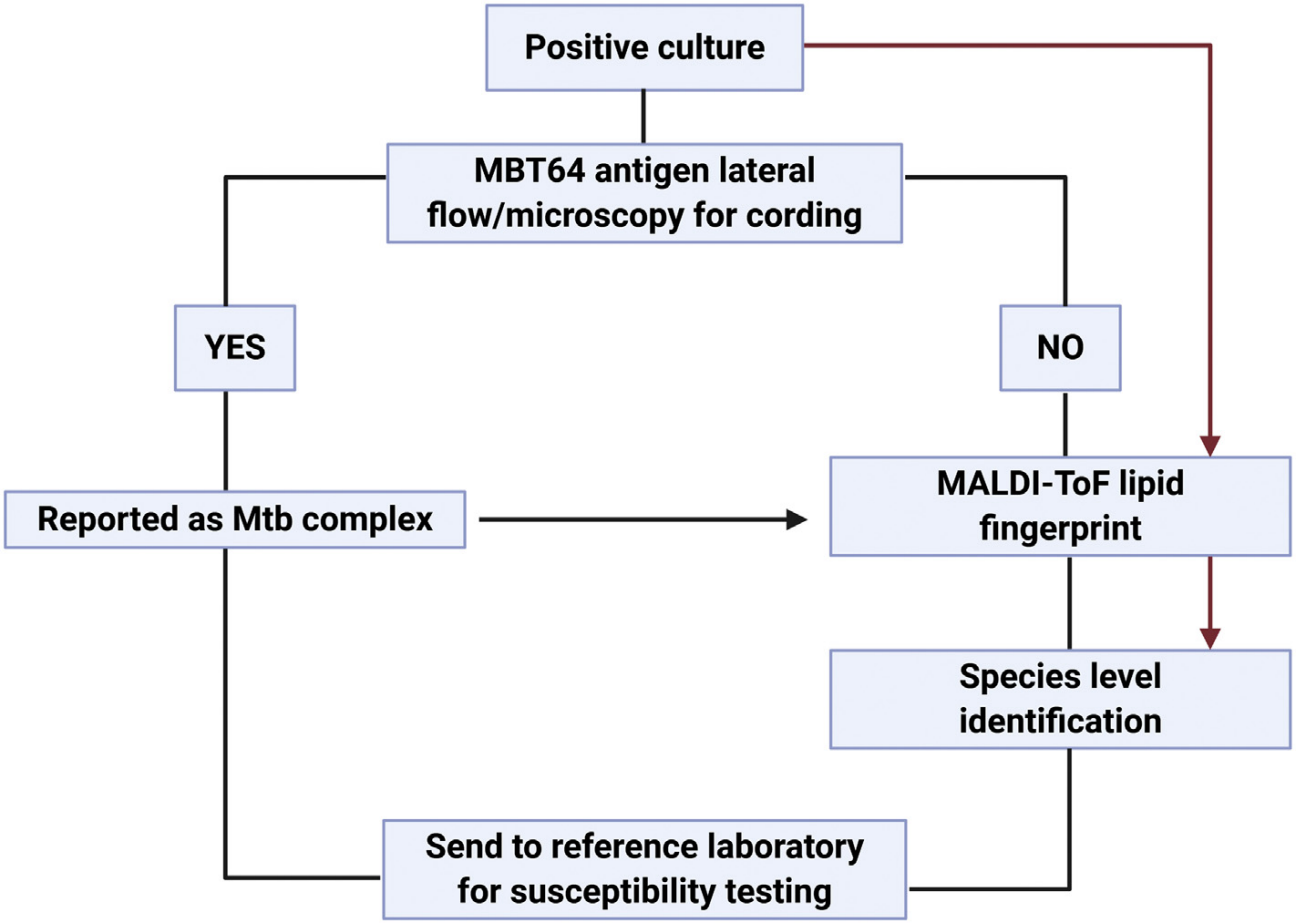
Proposed flowchart. With modern clinical matrix-assisted laser desorption/ionization time-of-flight (MALDI-ToF) systems delivering positive and negative ion modes (e.g. Bruker Biotyper Sirius), an inexpensive, rapid, direct pathway from mycobacterial culture to identification is possible (red arrows). For systems with positive ion mode only, an initial rapid TB antigen test can identify *Mycobacterium tuberculosis* complex (MTB) cultures with information on non-tuberculous mycobacteria (NTMs) by MALDI-ToF acting as additional confirmation.

The MALDI-ToF instrument used in this study offered the possibility of obtaining spectra in both positive and negative ion modes. Negative ion mode showed better definition for Mtb while positive ion mode was better for NTM isolates. The MALDI-ToF system is widely available, and has a low consumable costs [41]. A flowchart exploring how this method could be implemented in the laboratory can be found in Fig. 1.

Newer systems such as the Bruker Sirius will become the next generation of clinical microbiology systems operating in positive and negative ion modes. Currently, most clinical laboratories have systems which operate in positive ion mode only. Where this is the case, a combination of inexpensive rapid antigen testing (MBT64) combined with lipidomic MALDI-ToF analysis would provide definitive TB identification (TB or not TB, and likely NTM species) rapidly and cheaply in all clinical laboratories and provide species-level identification for NTMs which can have immediate management implications in certain populations (cystic fibrosis, non-cystic fibrosis bronchiectasis, cardiothoracic surgery, immunosuppressed individuals) [[42], [43], [44], [45], [46]].

This study has some limitations. Mtb complex subspecies, such as *M. africanum*, and the animal-adapted *M. caprae*, *M. bovis*, *M. microti*, *M. pinnipedii*, and ‘*M. canettii*’, as well as NTMs belonging to the *M. abscessus* complex for example, were not included. We plan further studies to confirm that such a species-specific lipid-based approach allows the identification and discrimination of other mycobacteria within the Mtb complex, distinguishing *M. chimera* from *M. intracellulare*, and ultimately providing the drug susceptibility profile of clinical isolates. For this last, a promising avenue would still be the analysis of lipids, as Lahiri and colleagues reported that rifampicin-resistant mutations are associated with altered concentrations of mycobacterial lipids such as SL-I [47]. Future work will ascertain whether this study can be reproduced using instruments available in routine microbiology laboratories with the same matrix (super-DHB) but a less toxic solvent such as ethanol instead of the chloroform used here. This study was conducted with a MALDI mass spectrometer operating in both positive and negative ion mode, which not all clinical microbiology laboratories have access to at the moment. However, as the next generation of clinical microbiology MALDI-ToF identification systems will likely utilize both positive and negative ion modes to increase diagnostic potential (e.g. Bruker Biotyper Sirius), mycobacteria can be identified with confidence as MTB complex or NTM rapidly, safely and inexpensively. The newer MALDI-ToF systems will increase the reliability of NTM species and subspecies identification. As part of this process, automated software based on the lipid fingerprint of each mycobacterium should be implemented for rapid identification and to simplify data analysis. Indeed, we are a step closer to the possibility of detailed identification to species level and detection of significant antibiotic resistance from a single spectrum in any clinical microbiology laboratory.

## Author contributions

XG, AB, FD and GL-M designed and performed the experiments. XG, AB, FD and GL-M analysed the data. All co-authors contributed to writing the paper.

## Transparency declaration

FD and GL-M are co-inventors of the rapid detection of mycobacteria based on species-specific lipids, for which a patent has been filed by Imperial Technology Transfer. The remaining authors have no conflicts of interest to declare. This study was supported by the 10.13039/501100007155MRC Confidence in Concept Fund and the ISSF Wellcome Trust Grant 105603/Z/14/Z (to GL-M and FD).
