# Supplementary material for: Performance of lipid fingerprint-based MALDI-ToF for the diagnosis of mycobacterial infections
Source: Clin Microbiol Infect. 2021 Jun;27(6):912.e1–5. doi: 10.1016/j.cmi.2020.08.027 (PMC8186428; doi:10.1016/j.cmi.2020.08.027)
Supplement: Multimedia component 1 [file mmc1.docx]

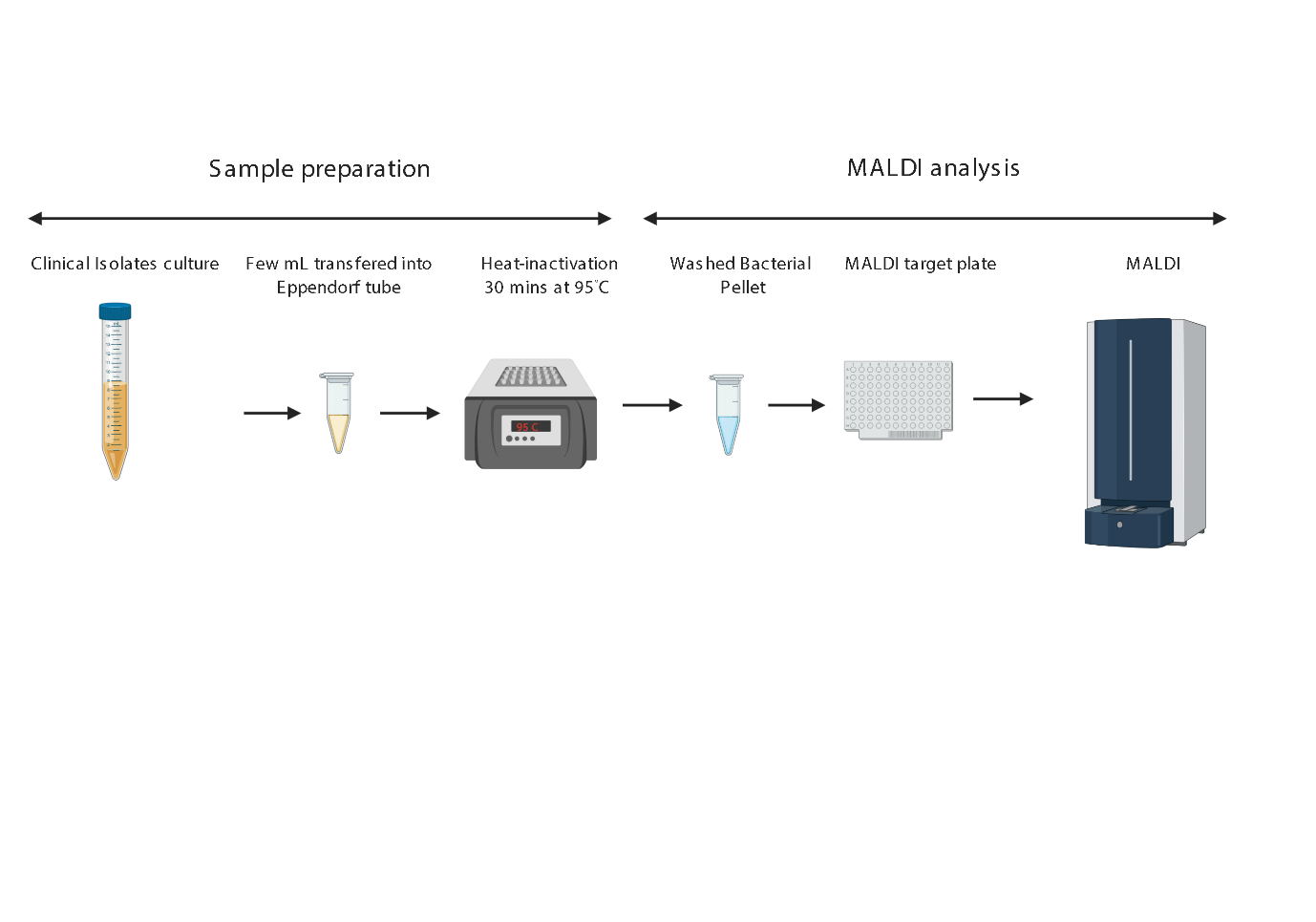


**Supplementary Figure 1: Schematic diagram of the sample preparation process for the test performed on the MALDI mass spectrometry system.** 100 μL of mycobacterial culture are aliquoted in an Eppendorf tube or screw-cap tube. The mycobacterial suspension is then heat-inactivated at 95°C for 30 minutes. The heat-inactivated mycobacteria are the washed 4 times with double-distilled. 0.4 μL of this preparation are loaded into the MALDI target plate followed by the addition of 0.8 μL of the matrix (super-DHB solubilized at 10 mg/mL in chloroform:methanol 9:1) and mixed on the MALDI target plate. Once dried, the mass spectra are acquired in the linear positive-ion and negative-ion mode. The image has been created with BioRender.
