# Supplementary material for: Performance of lipid fingerprint-based MALDI-ToF for the diagnosis of mycobacterial infections
Source: Clin Microbiol Infect. 2021 Jun;27(6):912.e1–5. doi: 10.1016/j.cmi.2020.08.027 (PMC8186428; doi:10.1016/j.cmi.2020.08.027)
Supplement: Multimedia component 2 [file mmc2.docx]

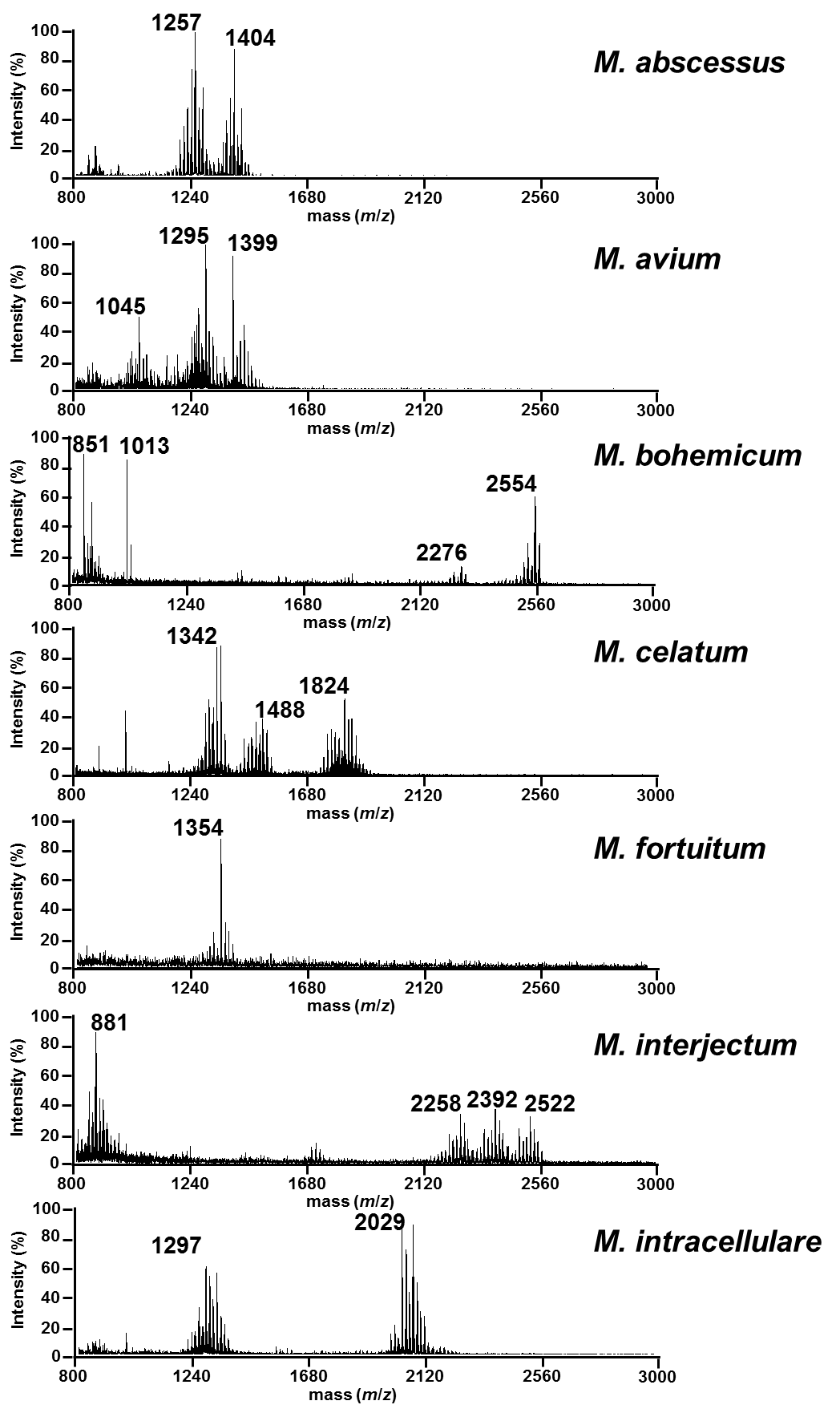


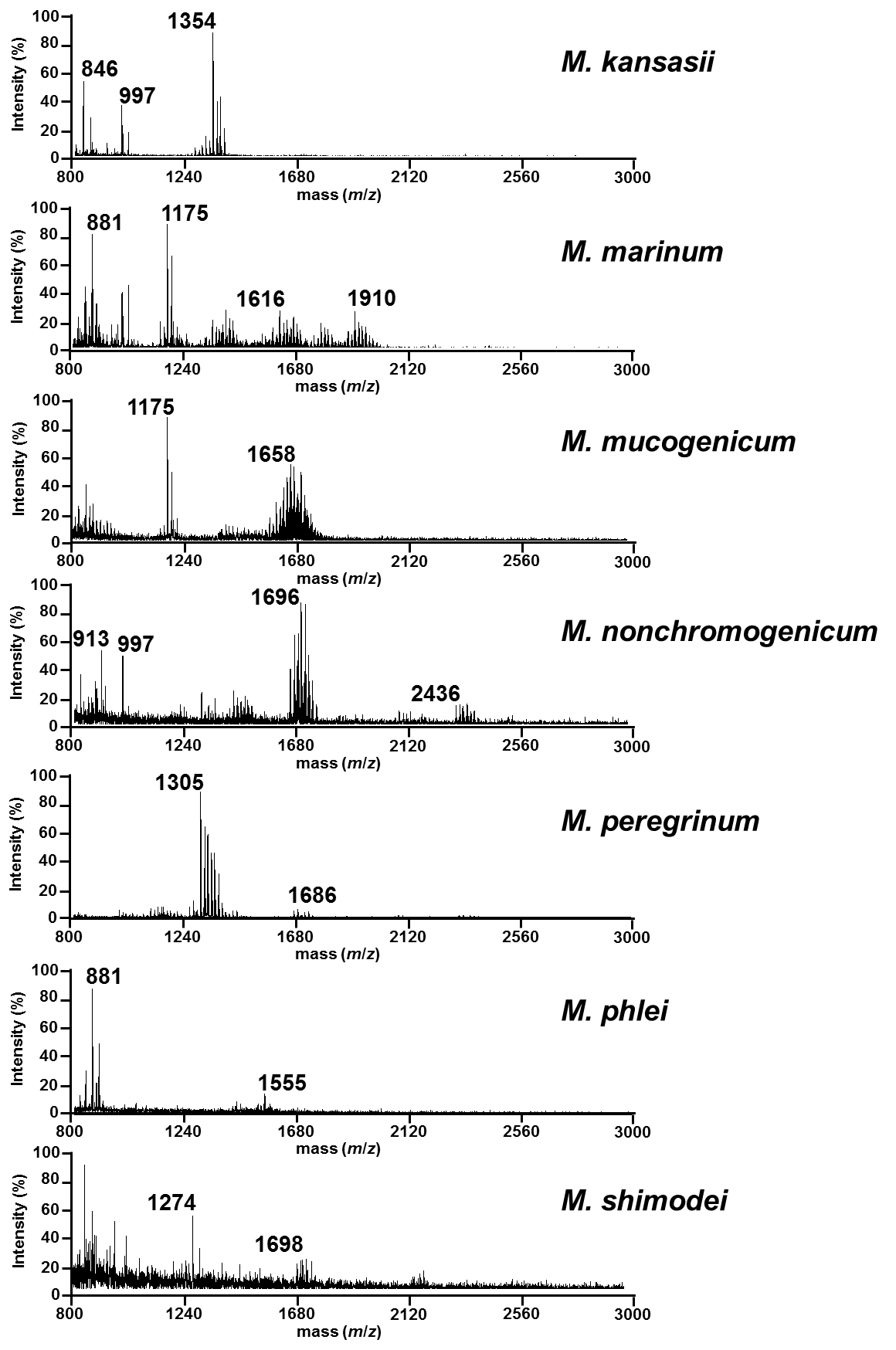


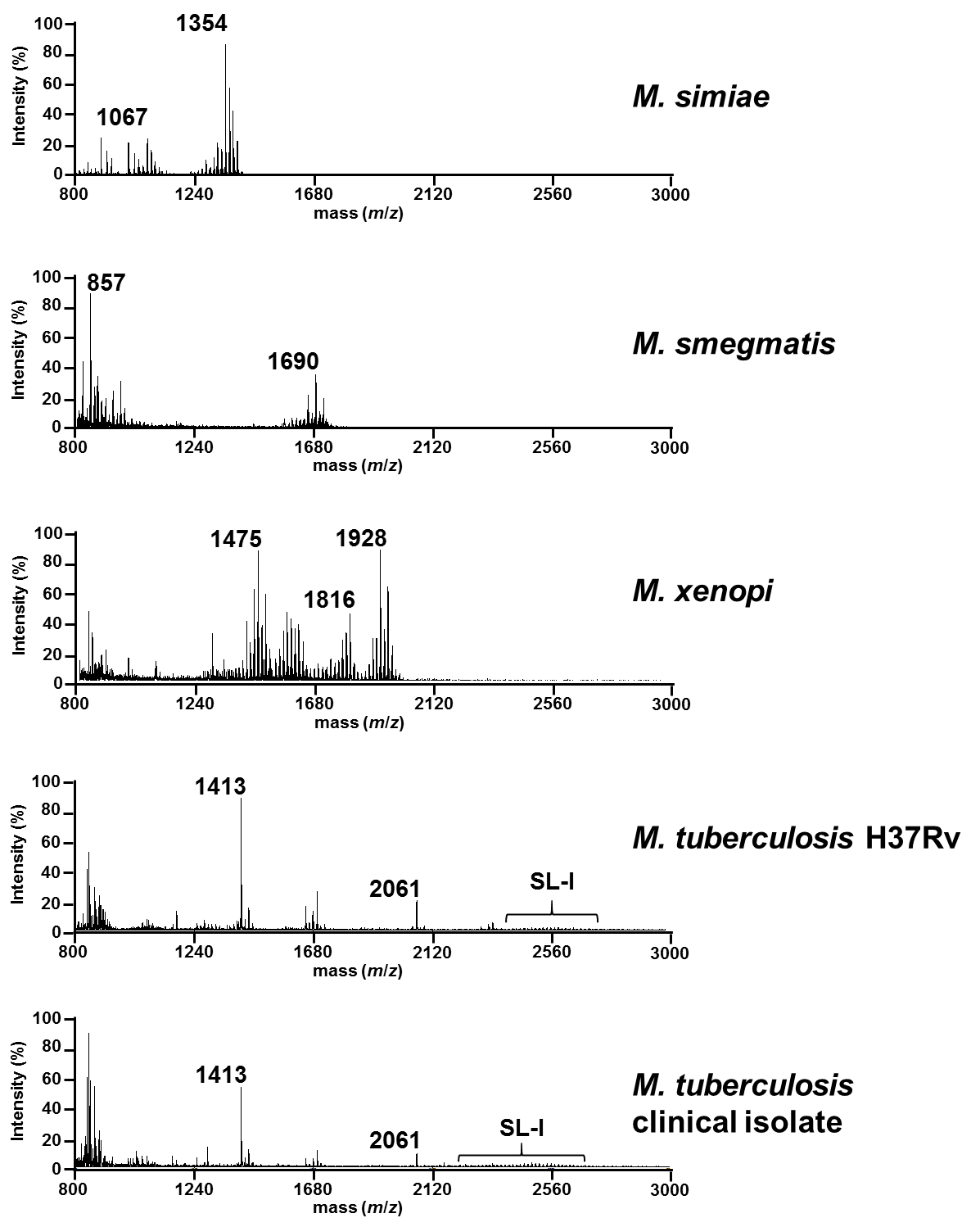


**Supplementary Figure 2**: Mass spectra of NTM clinical isolates in the positive ion mode and *M. tuberculosis* H37Rv and a representative of *M. tuberculosis* clinical isolates in the negative ion mode. Mass spectra were recorded from *m*/*z* 800 to *m*/*z* 3000.
