# Supplementary material for: Performance of lipid fingerprint-based MALDI-ToF for the diagnosis of mycobacterial infections
Source: Clin Microbiol Infect. 2021 Jun;27(6):912.e1–5. doi: 10.1016/j.cmi.2020.08.027 (PMC8186428; doi:10.1016/j.cmi.2020.08.027)
Supplement: Multimedia component 3 [file mmc3.docx]

**Supplementary Table 1. Schematic comparison of extraction methods**

| **Lipidomics sample preparation** | **Proteomics sample preparation** | **Proteomics quick sample preparation^11^** |
| --- | --- | --- |
| Positive culture  Heat-killed for 30 min at 95 °C  Pellet is washed with distilled water (distilled water added, vortex for 1 minute, centrifuge for 5 minutes, discard the supernatant and repeat 3 more times. After the last wash, re-suspend the pellet on 20 microlitres of distilled water)  Matrix and sample spotting | Positive culture  Heat-killed at 95 °C for 30 min  Pellet is washed with distilled water  Pellet is washed in 70% ethanol  70% formic acid is added to the pellet  Zirconia/silica beads are added.  Tube is vortexed for 10 min.  Addition of 100% acetonitrile.  Tube is vortexed for 10 min. Supernatant was utilized for analysis  Matrix and sample spotting | Positive culture  A loopful inoculated into 200μL of extraction solution (70% formic acid and 100% acetonitrile 1:1) with diameterzirconia/silica beads  Incubation at room temperature for 5 min to kill the mycobacteria  Proteins extracted by disruption of the organisms in a PowerLyzer24 high power bead-based homogenizer (Mo Bio Laboratories, Inc.) with 2 cycles of 45 s at 4,000 rpm with a 30-s rest interval in between  Samples centrifuged at 13,000 rpm for 1 min  1μL of extract is directly spotted onto a target plate for MALDI-ToF MS analysis.  For the swab-rapid extraction method developed for limited biomass of the slowly growing mycobacteria, a swab moistened in sterile water is used to obtain mycobacteria from the Middlebrook 7H11 agar into 500μL of sterile water in a 1.5-mL screw-cap tube  The samples are centrifuged at 13,000 rpm for 2 min  The pellets are resuspended in the extraction solution with zirco-nia/silica beads and vortexed briefly, and then protein is extracted as a loop-rapid extraction method |
